# Supplementary material for: Ambient temperature and cardiovascular mortality: a systematic review and meta-analysis
Source: PeerJ. 2017 Aug 4;5:e3574. doi: 10.7717/peerj.3574 (PMC5546177; doi:10.7717/peerj.3574)
Supplement: Supplemental Information 1 [file peerj-05-3574-s002.docx]

In many primary studies, we found many controversial data about the climatological effect on risk of cardiovascular (CVD) mortality. In many of these studies, results showed cold temperature had a strong effect on CVD mortality and in another study, it had the opposite effect.The Study on different geographical location had different data.

Although previously Turner et al conducted a systematic review and meta- analysis in this scope, but they investigated ambient temperature and cardiorespiratory morbidity([Turner et al. 2012](#_ENREF_2)). Another systematic review in this relation done by Bahaskaran et al they evaluated ambient temperature and incidence of myocardial infarction([Bhaskaran et al. 2009](#_ENREF_1)).

These contradictions in primary studies and lack of a systematic review and meta- analysis that show effects of ambient temperature on cardiovascular mortality was the motivation of this study. The best Knowledge of our study exactly showed that effect of cold and hot temperature on risk of cardiovascular mortality in general, according to gender and old person separately. Also, we provided many valuable data about the effect of living area on this risk.

Bhaskaran K, Hajat S, Haines A, Herrett E, Wilkinson P, and Smeeth L. 2009. The effects of ambient temperature on the incidence of myocardial infarction–A systematic review. *Heart*.

Turner LR, Barnett AG, Connell D, and Tong S. 2012. Ambient temperature and cardiorespiratory morbidity: a systematic review and meta-analysis. *Epidemiology* 23:594-606.
